# Recent Advances in Electrospinning Techniques for Precise Medicine

**DOI:** 10.34133/cbsystems.0101

**Published:** 2024-05-22

**Authors:** Wei Li, Yue Yin, Huaijuan Zhou, Yingwei Fan, Yingting Yang, Qiqi Gao, Pei Li, Ge Gao, Jinhua Li

**Affiliations:** ^1^ School of Medical Technology, Beijing Institute of Technology, Beijing 100081, China.; ^2^ Zhengzhou Academy of Intelligent Technology, Beijing Institute of Technology, Zhengzhou 450040, China.; ^3^ Advanced Research Institute of Multidisciplinary Sciences, Beijing Institute of Technology, Beijing 100081, China.; ^4^ Center for Advanced Biotechnology and Medicine, Rutgers University, Piscataway, NJ, USA.

## Abstract

In the realm of precise medicine, the advancement of manufacturing technologies is vital for enhancing the capabilities of medical devices such as nano/microrobots, wearable/implantable biosensors, and organ-on-chip systems, which serve to accurately acquire and analyze patients’ physiopathological information and to perform patient-specific therapy. Electrospinning holds great promise in engineering materials and components for advanced medical devices, due to the demonstrated ability to advance the development of nanomaterial science. Nevertheless, challenges such as limited composition variety, uncontrollable fiber orientation, difficulties in incorporating fragile molecules and cells, and low production effectiveness hindered its further application. To overcome these challenges, advanced electrospinning techniques have been explored to manufacture functional composites, orchestrated structures, living constructs, and scale-up fabrication. This review delves into the recent advances of electrospinning techniques and underscores their potential in revolutionizing the field of precise medicine, upon introducing the fundamental information of conventional electrospinning techniques, as well as discussing the current challenges and future perspectives.

## Introduction

Precise medicine, a novel healthcare paradigm based on clinical diagnosis and analysis of patients biological information, has emerged as a healthcare innovation since it provides disease diagnosis, prevention, and treatment that take into account individual variability in genes, environment, and lifestyle for each patient [1–3]. In contrast to the conventional “one size fits all” clinical and healthcare, precise medicine allows doctors and researchers to predict, design, and implement optimal or specific treatments for particular patients. To achieve this goal, it is critical to acquire and analyze sufficient biological and physiopathological information from patients and utilize instructive medical data to realize patient-specific therapy. Advanced devices for healthcare such as medical nano/microrobot, wearable/implantable biosensors, and human organ-on-chips (OOCs) have emerged in recent years, allowing for the target delivery of drugs/cells, precise monitoring of physiological conditions, and evaluation of patients’ responses toward customized pharmaceutic combinations [4–8]. Despite this, the design and manufacturing of these sophisticated instruments necessitate innovations in engineering techniques.

As an important direction of modern biomedicine, the medical microrobots are functional devices that can perform navigated motions in the human body driven by either extrinsic stimulations (e.g., light/magnetic/sonic field, pH, and chemicals) or self-propulsion (e.g., mobility of cells and microorganisms) to realize microscopic treatment [9–12]. These tiny surgeons have contributed to advancing targeted therapy, less/noninvasive medical surgery, and medical imaging [13–15]. Currently, a variety of techniques, including physical vapor deposition, laser direct writing 3-dimensional (3D) printing, electrochemical deposition, and wet chemical synthesis, have been utilized to manufacture microrobots. However, these prevalent techniques are limited to stably and massively producing functional microrobots at low cost, which should control the robots’ dimensions and structures and integrate stimuli-responsive materials or biological components to realize intelligent manipulations.

Diagnosis and analytics are crucial processes in precise medicine. Without accurate and in-time detection of abnormal physiological phenomena presenting in individuals, it is challenging to provide appropriate and effective medical treatments. Wearable and implantable biosensors are capable of monitoring the status of patients by sensing the changes in target analytes via biological components (bioreceptor or biomolecule) that are subsequently converted to measurable signals (e.g., electrical, optical, thermal, or other forms) for real-time readouts [16,17]. Considering the required effectiveness and working environment, an eligible biosensor should possess high sensitivity that can precisely discern subtle physiological changes, as well as excellent biocompatibility, flexibility, and/or long-term in vivo stability for user acceptance. Hence, advanced materials and components with sensing performance and tunable physiochemical properties are truly needed to facilitate the development of biosensors.

The emergence of OOCs paved the way to create a miniature human tissue/organ model on a compartmentalized microfluidic chip. By orchestrating multiple types of cells and supplying physiological signals [e.g., dynamic mechanical forces, extracellular matrix (ECM) cues, and biochemical gradients], the OOCs provide a more relevant physiological recapitulation compared with animal and planar cell models. More importantly, with the involvement of the cells isolated from patients, it is possible to use such devices to establish patient-specific pathological microenvironments that offer benefits in the exploration of personalized treatment such as optimization of drugs dosage and combination. While the OOCs have shown great promise, continued advancements in materials and fabrication techniques for improving the sophistication and reliability will likely enhance the biomimetic reflection of such platforms thereby broadening their applications in precise medicine.

Electrospinning is a broadly used nanotechnology that obtains continuous fibers from polymer solutions or melts in an electrostatic field, which has the advantages of scalability, wide material adaptivity, ease of functionalization, and low cost [18–20]. By regulating processing parameters, this technique allows for the production of various functional materials and structures, such as nanofibers, microbeads, and porous membranes that have been widely used as biomedical products [9,21–23]. Nevertheless, several critical drawbacks such as chaotic fiber deposition, lack of multiple functional integrations, and low production effectiveness have hindered its translation from bench to bedside. Most importantly, toxic solvents widely used in the electrospinning process usually confine the involvement of biological elements such as biomolecules and living cells within the fabricated nano/microfibers, which substantially limits its further applications in precise medicine.

To overcome these challenges, pioneering efforts have been made to define the fiber patterns, enrich the composition of fibers, realize large-scale production, and enclose vulnerable molecules and cells, thus bringing in diverse advanced electrospinning techniques [20,24,25]. Upon leveraging these merits of electrospinning, advanced building blocks could be designed, manufactured, and incorporated with medical microrobots, biosensors, and OOCs to promote their performances and functional diversity for achieving enriched applications in precise medicine.

**Fig. 1.**
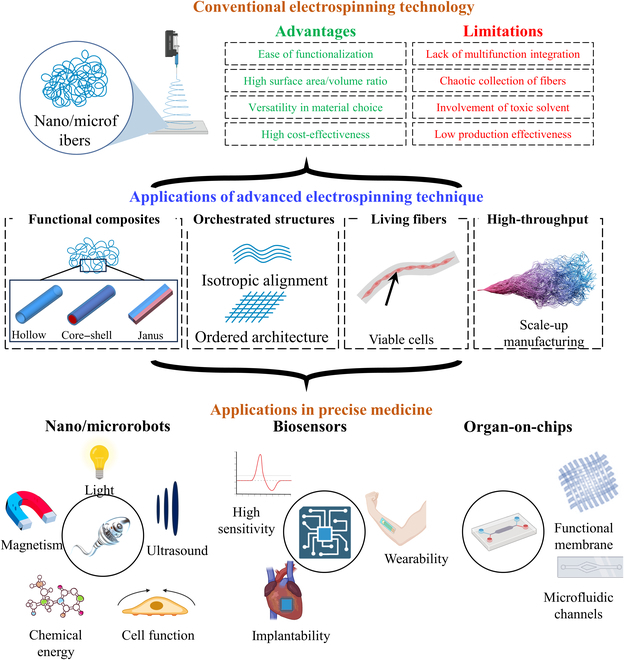
Schematic overview of the review. This figure was created using materials from BioRender.

This review focuses on the recently emerged advanced electrospinning techniques that are categorized into the manufacturing of functional composites, orchestrated structures, living constructs, and high-throughput manufacturing fibers, discussing their impacts on improving the performances of biomedical devices, including medical microrobots, biosensors, and OOCs, and current challenges are discussed along with future perspectives (Fig. 1).

## Overview of Electrospinning

### Working principle of electrospinning

**Fig. 2.**
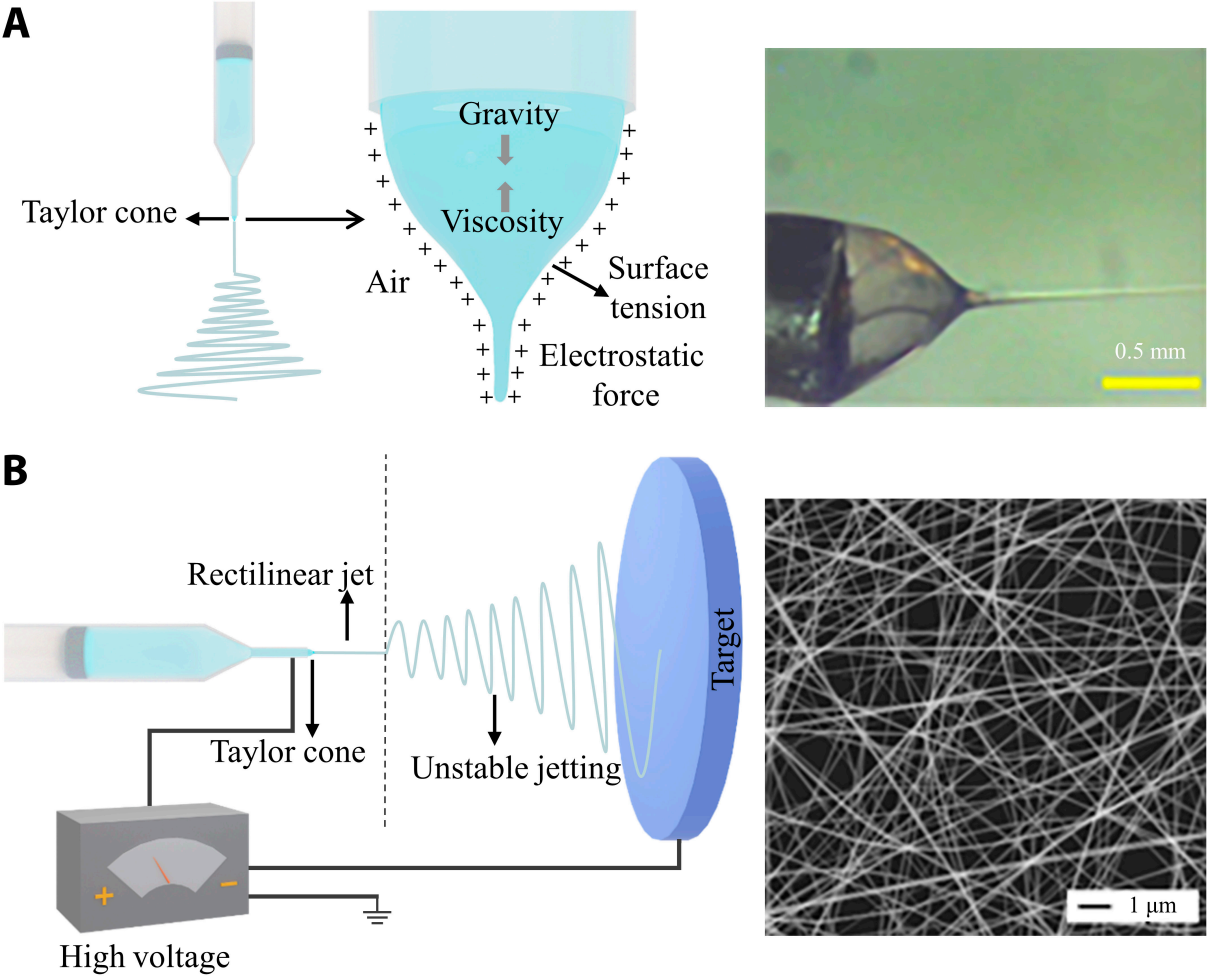
Schematic of electrospinning setup and process. (A) Schematic diagram of the formation of the Taylor cone and the screenshots of the Taylor cone. Reproduced with permission from [28]. Copyright 2020 American Chemical Society. (B) Schematic diagram of electrospinning and the image of the electrospinning fibers. Reproduced with permission from [35]. Copyright 2003 American Chemical Society.

Electrospinning is the manufacturing progress for the production of nano/microfibers, in which melts or polymer solutions spin fibers in the electric field created by a high voltage up to several tens of kilovolts [19]. The initiation of fibers relies on overwhelming the surface tension of the polymer solution by the electrostatic repulsion [21]. As the repulsive force is strong enough to overcome the surface tension, the fluid forms a taylor cone [26], which serves as the location of the ejection of an initial fiber [27]. Under the coupled fields of electrostatic force, surface tension, viscoelasticity, air resistance force, and gravity, the liquid produces a jet that is subsequently stretched to generate ultrafine fibers (Fig. 2A) [28]. As the solvent evaporates, the fibers are solidified and stacked onto a designed collector (e.g., roller and plane) to obtain different structures such as tubes and membranes [29]. The jetting process in electrospinning mainly includes 3 stages: the formation of the Taylor cone, rectilinear jetting, and unstable jetting [30]. The rectilinear jetting usually occurs in the initial stage of jetting, where the jet is in a straight segment [31,32]. During the unstable jetting stage, the jet stretches rapidly, forming a spiral, bending path, and the fiber diameter decreases rapidly (Fig. 2B) [33–36].

**Table.** Comparison of advantages and disadvantages of electrospinning technology [26,76]

Category	Technique	Advantages	Disadvantages
**Functionalized composites**	Multiaxial electrospinning	· Core–shell fibers	· Complex setup and processing
		· Hollow fibers	
		· Porous fibers	
		· Improved material adaptability	
	Emulsion electrospinning	· Simple processing	· Complex preparation progress of emulsion
		· Fine fiber	
		· Improved material adaptability	
	Conjugated electrospinning	· Janus fibers	· Requires complex setup and special collection manner
	Chaotic electrospinning	· Multiscale stratified fibers	· Difficult to preservation of micro/nanostructures
**Orchestrated structures**	Near-field electrospinning	· Tunable fiber alignment	· Difficult to make nanoscale fibers
		· Wide range of applicable materials	
	MEW	· Tunable fiber alignment	· Only applicable to molten polymers
		· Longer linear jetting distance	
**Living fibers**	CE	· Direct embedding living cells	· Strict requirements for mild parameters
		· Guided cell alignment and functions	
**High-throughput manufacturing**	Needleless electrospinning	· High efficiency	· Unstable jet
		· Avoiding needle blockage	
	Centrifuge electrospinning	· High efficiency	· Fiber collection is difficult
		· Controllable diameter	

### Materials for electrospinning

Back to the early of 20th century, the electrospinning was originally developed to create textiles and filters using the electrically stretched fine polymer fibers [37]. With the advent of advanced materials science and nanotechnology, a variety of functional materials, such as conductive polymers, stimuli-responsive materials, and biomaterials (e.g., hydrogels, cellulose acetate [38], and proteins) were utilized to electrospin ultrafine structures, which greatly expanded its application in tissue engineering, biosensor, and drug delivery. In general, the materials for electrospinning can be categorized into synthetic and natural materials.

Synthetic materials are formulated from polymer compounds that can possess ideal mechanical properties by regulating molecular weight and concentration. As an acceptable material for electrospinning, the polymer should meet several critical requirements, including appropriate electrical conductivity and solubility or meltability. To be applied in biomedical scenarios, the electrospun materials are expected to be biocompatible and nontoxic. Polycaprolactone (PCL) is a representative electrospun material for biomedical applications (e.g., drug release systems and regenerative materials) due to the great biocompatibility stemming from the nontoxicity and safe degradation products (merely carbon dioxide and water) [39]. Polylactide, polyurethane (PU), and other biocompatible polymers are also commonly used biomaterials for electrospinning of tissue engineering products [40]. In addition, stimuli-responsive materials (also known as smart materials), which exhibit property changes in response to external stimuli including temperature, pH, light, magnetic/acoustic fields, and biochemical reagents, have been used to electrospun nano/microfibers. In combination with the advantage of nanomaterials, these materials enable the creation of fibers or fabrics with unique and adaptable characteristics, which accelerate the development of modern medical devices.

Natural materials are derived from creatures, encompassing a rich variety of proteins, polysaccharides, glycosaminoglycans, etc. [41]. These materials resemble the structural and compositional features of ECM and thus have superior bioactivity and biocompatibility to synthetic materials. Chemical solvents enable the electrospinning of natural polymers such as collagen, gelatin, chitosan, etc. When exposed to solvents, the structures of proteins might be changed, resulting in damage to their biofunctionalities. Therefore, to protect the natural polymers, mild aqueous solutions (e.g., saline, etc.) are preferable for their electrospinning. Since the viscosity and conductivity of the aqueous solution are inferior to those of organic solvents, the electrospinning of natural materials alone remains challenging [36]. Only a few numbers of natural polymers residing in aqueous agents have been used to produce electrospun fibers. Alginate is a natural polysaccharide widely used in electrospinning [42,43]. This material can be modified to adjust its solubility, hydrophobicity, and biological activity [44,45]. However, alginate aqueous solution is difficult to electrospin due to its high surface tension and high electrical conductivity. The addition of other polymers with highly tangled molecules [e.g., polyethylene oxide (PEO)] can effectively overcome these limitations by reducing the electrical conductivity and surface tension of the alginate solution, which allows the successful formation of electrospinning fibers.

### Advanced electrospinning technologies

Undoubtedly, electrospinning owns a unique capacity to produce structures with ultrafine fibers and high surface area-to-volume ratio at low cost. Nonetheless, the conventional method is limited in integrating different types of materials to produce composite fibers and complex or highly ordered structures and thus is difficult to meet the demands of precise medicine. Besides, the toxic risk of involved organic solvent and low scalability also hindered its applications and commercialization in the field of healthcare. To overcome these challenges, continuous efforts have attempted to explore advanced electrospinning techniques, which are summarized in this section.

#### Electrospinning of functionalized composites

Conventional electrospinning can only utilize a single material to manufacture solid nano/microfibers, which limits both the compositional and structural features of the resultant constructs. The application of innovative electrospinning techniques leads to the integration of multiple types of materials with different properties during the electrospinning process, giving rise to heterogeneous fibers with diverse structures and functionalities (e.g., core–shell, follow, porous, and Janus fibers) [46,47].

**Fig. 3.**
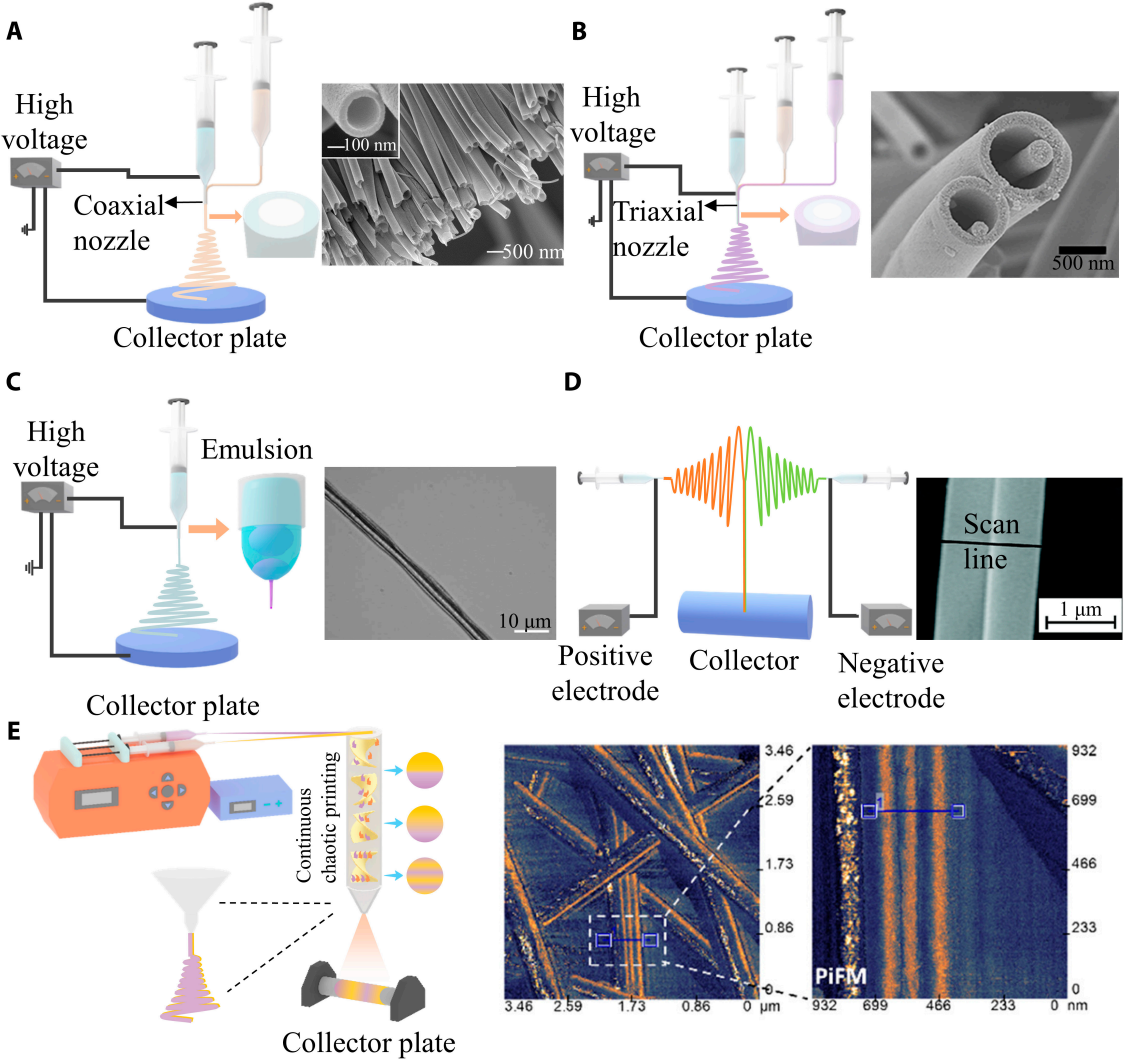
Schematic of advanced electrospinning for heterogeneous fibers. (A) Schematic of coaxial electrospinning and fibers that are from scanning electron microscopy (SEM). Reproduced with permission from [48]. Copyright 2004 American Chemical Society. (B) Schematic of triaxial electrospinning and SEM observation of triaxial fibers. Reproduced with permission from [58]. Copyright 2010 American Chemical Society. (C) Schematic of emulsion electrospinning and image of core–shell fiber. Reproduced with permission from [66]. Copyright 2007 American Chemical Society. (D) Schematic of conjugated electrospinning and SEM image of the fiber. Reproduced with permission from [50]. Copyright 2019 The Royal Society of Chemistry. (E) Schematic of chaotic electrospinning and micrographs of chaotic electrospinning fibers. Reproduced with permission from [76]. Copyright 2021 American Chemical Society.

To realize the production of core–shell fibers, the single spinneret is usually replaced by a coaxial nozzle consisting of an outer and inner nozzle. Two needles are connected to 2 different solution reservoirs (Fig. 3A)[28,48–50]. The coaxial nozzle can simultaneously electrospin 2 types of materials, obtaining either composite fibers manifesting the properties of integrated components or hollow fibers with tunable microstructures (e.g., channel diameter and wall thickness). This technique enables the involvement of the unelectrospinnable or vulnerable materials (e.g., hydrogel [51], nonconductive polymers [52], cells [53], and enzymes [54]) by encapsulating them in the core of an ultrafine composite fiber, extensively broadening the scope of applicable materials. Besides, with the copresence of multiple materials, the fabricated composite can integrate the advantages of residing payloads, exhibiting unparalleled performances that are difficult to achieve by individuals. For instance, gelatin is a water-soluble biomaterial that is a challenge to electrospun because of its high surface tension, which causes the polymer jets to become unstable and form droplets [55]. Polymers with good spinnability, such as PCL, polyvinyl alcohol (PVA), and PEO, have been used as a coaxially electrospun sheath to realize the fabrication of gelatin-based nanofibers. The composite fibers consist of natural and synthetic polymers that achieve both good cytocompatibility and robust mechanical strength, thereby being used in tissue repair and regeneration. As another representative example, the hollow and the core-sheath electrospun fibers can be used for controlled drug delivery [56]. Compared with the single fibers, the drug loaded in the hollow and the core-sheath fiber can avoid a burst release when exposed to the fluidic environment in the human body [57].

As an update of coaxial electrospinning, the triaxial electrospinning can simultaneously use 3 polymer solutions to manufacture structurally complex fibers (Fig. 3B) [58–60]. The spinneret is divided into a core, an inner shell, and an outer shell. Notably, for the 3 types of materials used for multiaxial electrospinning, only one fluid should be spinnable to enable the electrospinning process, while the others can easily go with the flow [61]. Therefore, the combination of materials immensely expands the selection of electrospinning materials and is beneficial to the development of advanced electrospinning products [62,63]. The improved material adaptability permits the development of multifunctional fabrics by the integration of different types of materials with distinct properties. For example, in a recent study, Wang et al. [64] designed and fabricated a high-performance microbelts and array with fluorescent display as well as tunable conduction and magnetism by coelectrospinning of 3 materials, CoFe_2_O_4_/polymethyl methacrylate (PMMA), polyaniline (PANI)/PMMA, and Tb(acac)3bpy/PMMA through a triaxial nozzle. The function of each layer can be regulated by adjusting the position and content of the 3 functional materials. Such a one-pot manufacturing of the triaxial microbelts not only retains the advantages of nano/micromaterial but also imparts multiple desired properties, thereby holding a great promise in various fields, such as microintegrated circuits, microchips, and micro/nanodevices [64]. Besides, relying on the triaxial electrospinning, different types of drugs can be compartmentally encapsulated in a nano/microcarrier that helps to achieve the spatiotemporal control of drug release kinetics [65]. Notwithstanding, because of the complexity of the setup and processing, the application of triaxial electrospinning technique is less extensive than the coaxial electrospinning method.

Emulsion electrospinning is another manufacturing technique for producing the composite fibers (Fig. 3C) [66]. Different from the coaxial ejection manner, emulsion electrospinning relies on the high viscosity and rapid evaporation of the solution in the outer layer. Under the electric field, emulsion droplets move from the surface to the center. Under the combined actions of gravity, surface tension, and coulomb forces, the droplets can form core–shell structure fibers [67,68]. The emulsion solutions are generally 2 immiscible liquids, water-in-oil or oil-in-water emulsion [69]. The use of mild solutions allows for the synthesis of functional materials with the encapsulation of fragile payloads, such as peptides [70], enzymes [71], and proteins [72]. In addition, since only one fluid is used, the emulsion electrospun fibers are generally thinner than those produced by coaxial electrospinning. Su et al. [73] used both coaxial and emulsion electrospinning to produce novel composites based on poly(epsilon-caprolactone)/PEO loaded with hemagglutinin and keratin for wound healing. By encapsulating drugs within the core of core–shell fibers, effective prevention of drug absorption during transportation is achieved. A shell layer with specific release characteristics enables controlled drug release kinetics. The material used for emulsion electrospinning must be an emulsified solution, which greatly limits the selection of electrospun materials. On the other hand, the presence of emulsified solution may adversely affect the quality of electrospun fibers [74].

Other than the coaxial integration, Janus nano/microfibers that are composed of 2 parallelly aligned units can be constructed using advanced electrospinning techniques. Conjugated electrospinning uses 2 high-voltage power supplies with opposite polarity to simultaneous electrospinning of 2 different materials from 2 spinnerets, aligning them side-by-side, forming a single fiber (Fig. 3D) [50]. The combination of 2 distinct materials enables the creation of composites with a plethora of functionalities. For example, Xi et al. [75] developed a highly anisotropic array film composed of electrospun Janus nanoribbons composed of conductive, magnetic, and fluorescent materials, which simultaneously possess anisotropically electrical conduction, superparamagnetism and fluorescence. Such a new Janus nanoribbon array film might become promising tool or component for the development of biosensors or diagnosis platforms.

To improve the complexity of the stratified composite fibers, chaotic electrospinning was recently suggested by Holmberg et al. [76]. Upon the chaotic printing technique that applies chaotic static mixers as printing nozzles to produce micrometer-sized filaments with internal multilayered structures by extrusion, chaotic electrospinning further imposes electrical fields to transit the dimensions of the filaments from the micro to the nanoscale (Fig. 3E) [76]. Using PVA and alginate, the structure composed of multiple-layered carbon nanofibers drastically increased the surface area, boosting its performance as electrodes for energy-storage applications (10-folds improvement compared with that manufactured by conventional electrospinning), such as supercapacitors.

#### Electrospinning of orchestrated structures

Conventional electrospinning gathers nanofibers that undergo the whipping stage, which results in the collection of disordered fibers. Therefore, aligned fibers with predesigned topology can endow the nanofibers with additional functions, such as isotropic mechanical improvement, guiding cellular migration, reinforcing conductivity, etc.

**Fig. 4.**
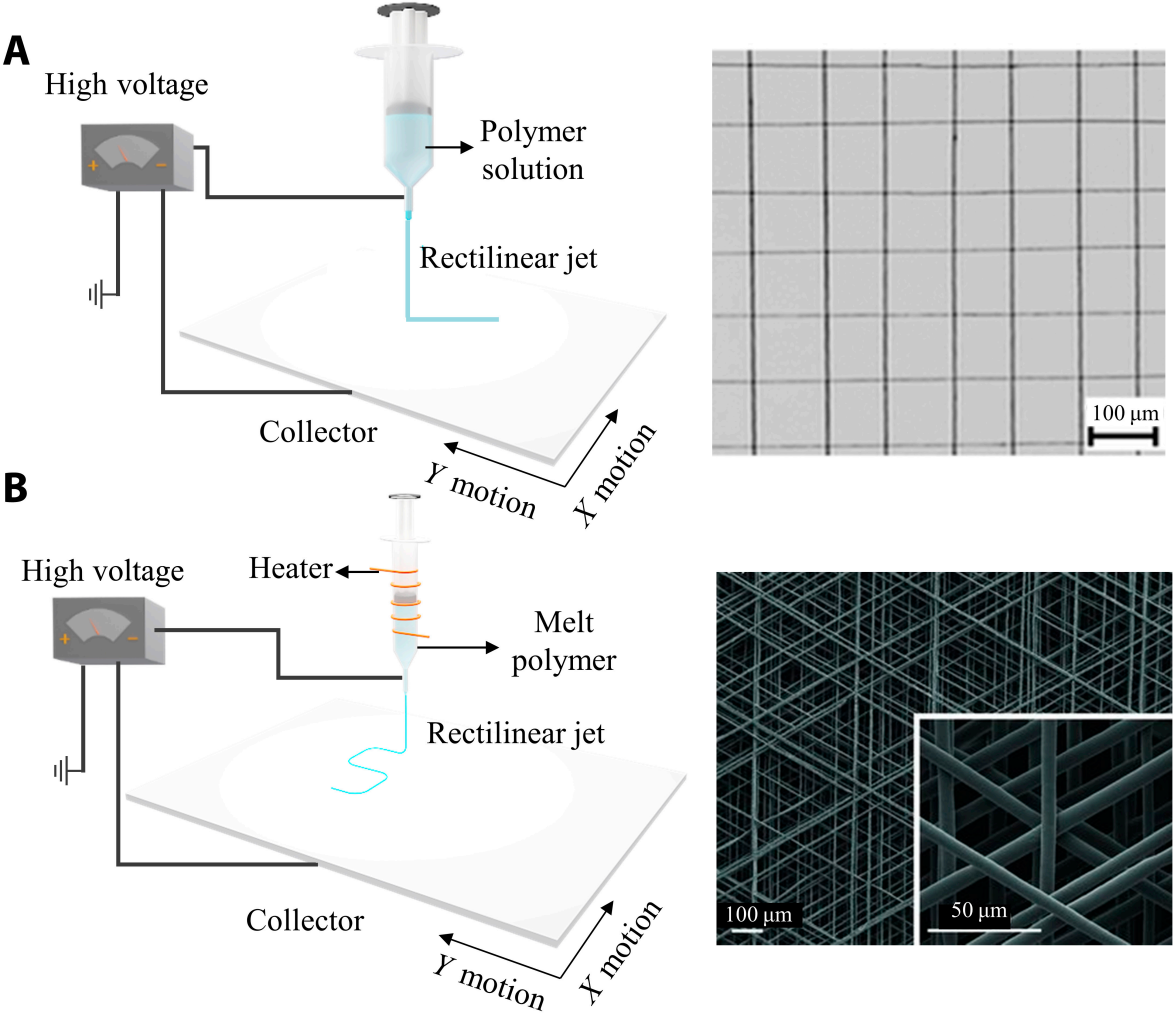
Schematic of advanced electrospinning for orchestrated fibers. (A) Schematic of near-field electrospinning and the fibers. Reproduced with permission from [79]. Copyright 2013 The Royal Society of Chemistry. (B) Schematic of melt electrospinning and the fibers. Reproduced with permission from [86]. Copyright 2011 WILEY-VCH Verlag GmbH.

Near-field electrospinning technology is proposed as a spinning method that can precisely control the fiber deposition site [77]. The principle of this technology is to control the spinning jet in the initial stable motion state by reducing the spinning distance (in the scale range of several centimeters or even several millimeters) and the spinning voltage (0.2 to 12 kV) to realize the precise control of the spinning jet and the precise deposition of the cured fiber [78]. Meanwhile, the collector is installed on a precise motion platform. By controlling the motion path, the electrospun fiber can be deposited at a specific point or according to a predetermined track in the *X*-*Y* plane, eventually obtaining a patterned structure (Fig. 4A) [79]. On the basis of this fiber-writing technique, membranes or scaffolds composed of isotopically aligned nano/microfibers or complex structures can be successfully manufactured, holding great potential for applications in microelectronics and tissue engineering [77,78,80,81]. Because of the decreased collection distance, the diameter of electrospinning fiber is significantly larger than that of traditional electrospinning. To minimize such adverse effects, the material solvent with high viscosity (such as PEO [82], PCL [83], polystyrene [84], and piezoelectric polyvinylidene fluoride [85]) may be more suitable for near-field electrospinning.

Melt electrowriting (MEW) is another manner for patterning the electrospun fibers. Similar to near-field electrospinning, MEW also operates in the region of the jet where the flight path is straight to achieve control over the deposition of electrospinning fiber (Fig. 4B) [86]. Unlike near-field electrospinning, this technique selects polymer melts as the materials instead of polymer solvents. The polymer melt generally possesses a higher viscosity and lower conductivity than the polymer solution, which can significantly elongate the straight jet path [86]. The stable area can extend to a few centimeters, and the diameter of the fiber (<100 nm to 500 μm [87]) will be smaller than the fibers made by near-field electrospinning (0.05 to 30 μm [78]). It is worth noting that the melt electrospinning requires material fusion to achieve structural integrity, which greatly limits the selection of electrospinning materials that should have high melting points.

#### Electrospinning of living fibers

Living components are one of the most important medical resources for regenerative and precise medicine. In particular, cell therapy, targeting deliver the genetically engineered cells or stem cells into the patients’ body, has demonstrated its unique advantages in tissue regeneration and disease treatment. Nonetheless, the injection of cell suspensions usually fails to realize target delivery for precise medicine. Therefore, embedding living cells within a tissue-engineered scaffold has been in the spotlight to further explore the power of cell therapy.

In recent decades, 3D bioprinting and microfluidics have been utilized to build the cell-laden bioconstructs. Despite this, their low resolutions (hundreds of micrometers) lead to difficulty in mimicking the features of the ECM. Hence, the fabricated constructs usually failed to govern cell activities and fates, such as cell alignment, organization, and tissue morphogenesis. On the other hand, despite the ability to produce ultrafine fibers, the conventional electrospinning process involves chemical solvents and high voltage, which inevitably damage cell viability. Therefore, it is necessary to innovate the electrospinning technique for realizing the direct incorporation of cells within nano/microfibers to engineered living constructs.

**Fig. 5.**
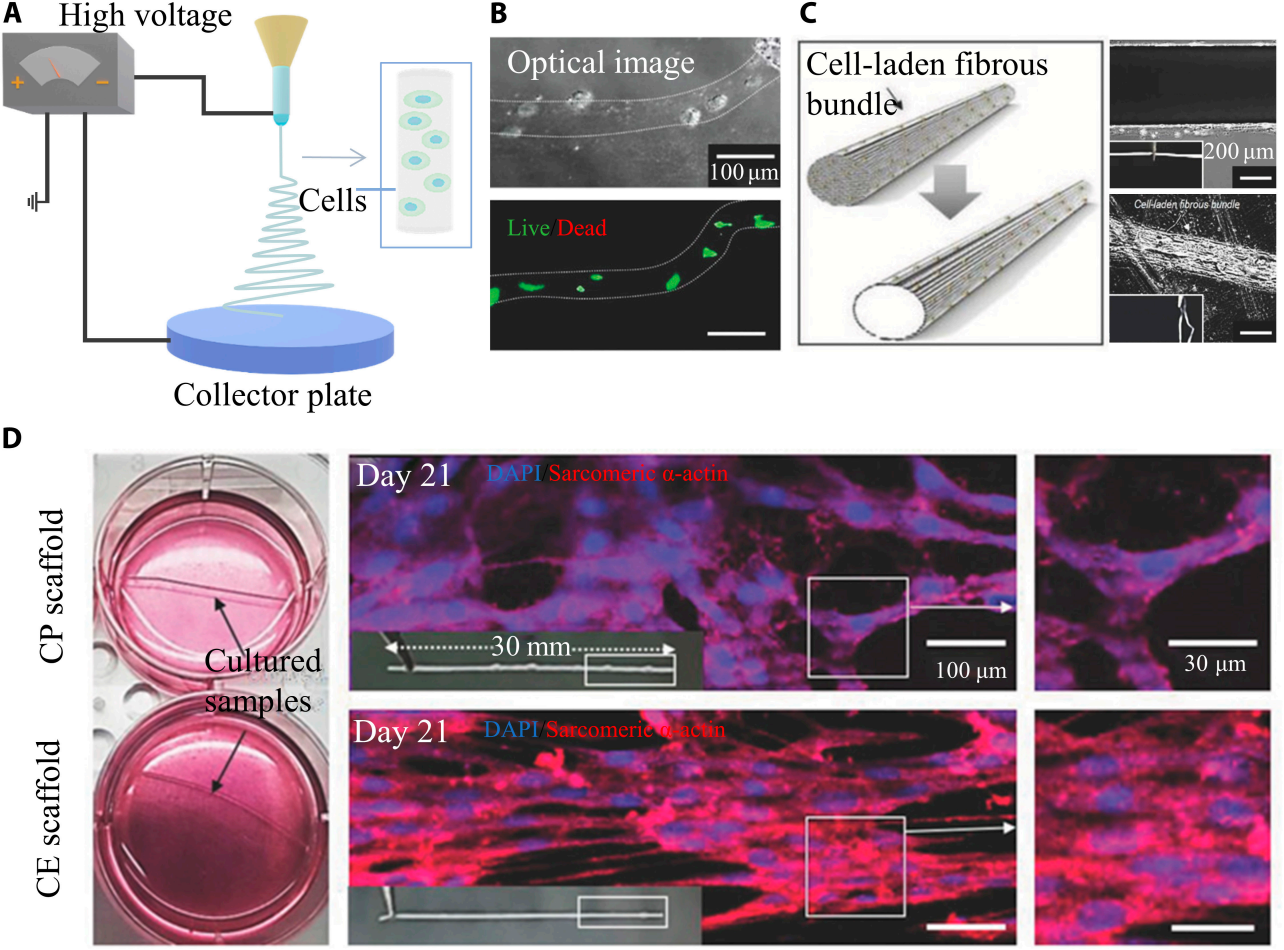
Schematic of advanced electrospinning for biofibers. (A) Scheme of electrospinning of biofibers. (B) The optical images and live/dead images of cell-laden electrospinning fibers. (C) Schematic and optical images of CE fibers. (D) Optical images and 4′,6-diamidino-2-phenylindole (DAPI)/sarcomeric α-actin staining images of CE and cell printing (CP) scaffold after 21 days of culture. Reproduced with permission from [53]. Copyright 2018 WILEY-VCH Verlag GmbH & Co. KGaA, Weinheim.

Several pioneering studies have attempted to electrospin cell-laden hydrogels to obtain biofibers. The key considerations of cell electrospinning (CE) include the selection of biomaterials for electrospinning and the processing parameters. While the material formulation should match both cytocompatibility and electrospinnability, the parameters such as voltage, electrical field intensity, and ambient humidity that might cause cell damage or apoptosis must be carefully designed. Yeo and Kim [53] used CE to successfully create and culture cell-laden alginate/PEO fibers (electric field intensity at 0.075 kV·mm^−1^, the distance between nozzle and electrode is 14 mm) Compared with cell printing scaffolds, CE scaffolds showed highly arranged, multinucleated cell morphology (Fig. 5). In their previous study [88], PEO/alginate composite fibers encapsulating C2C12 cells were electrospun to obtain a stable scaffold, in which the cells could survive and grow. In a recent report, Qiu et al. [89] performed microscale CE and successfully produced highly oriented structures. Therefore, previous efforts have overcome the challenge of directly incorporating cells in the electrospun fibers. Nonetheless, few studies have utilized the living fibers to engineer viable and functional bioconstructs for verifying the feasibility of tissue regeneration or targeted therapy, which might be attributed to the low manufacturing efficiency and limited scalability.

#### High-throughput manufacturing of nano/microfiber

Despite the numerous advantages of electrospinning technique and its products, the upscaling from laboratory-scale to industrial-scale production is the virtual challenge to commercialization and broadening applications of the electrospun materials. The most straightforward manner to solve this problem is scaling up the electrospinning using multineedle arrays. Besides, several advanced techniques, such as needless and centrifugal electrospinning techniques, are developed to realize the high-throughput manufacturing of nano/microfibers.

**Fig. 6.**
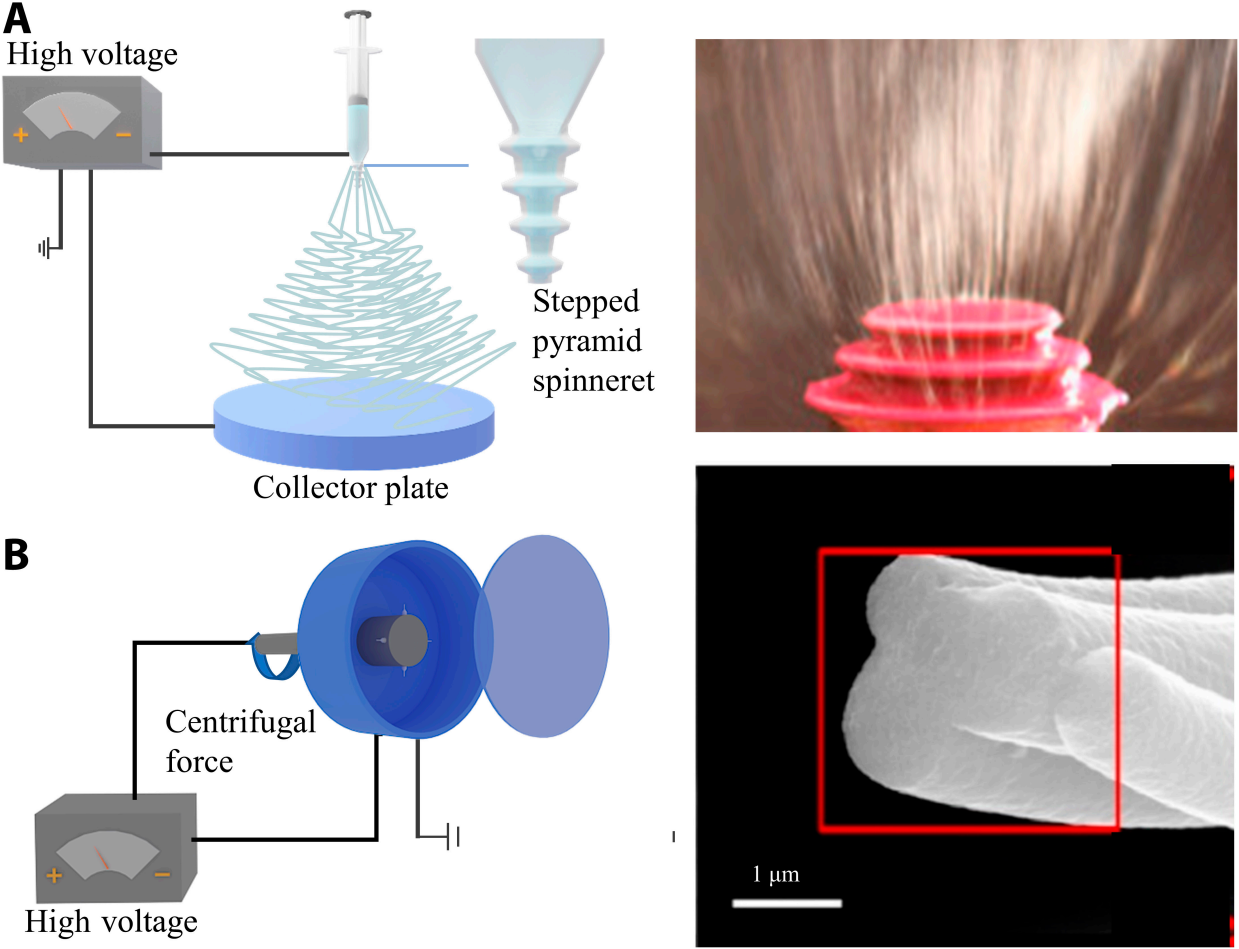
Schematic of advanced electrospinning for high-throughput manufacturing fibers. (A) Scheme of needleless electrospinning and the photograph of needleless electrospinning process. Reproduced with permission from [93]. Copyright 2015 Elsevier B.V. (B) Scheme of centrifuged electrospinning and the SEM images of the fiber made by centrifuged electrospinning. Reproduced with permission from [100]. Copyright 2021 Elsevier Inc.

In traditional electrospinning technology, the stability of the Taylor cone is difficult to control because of the multiple influencing factors (e.g., airflow disturbance, needle diameter, and needle blockage due to high viscosity). In addition, the existence of electrospinning needles also faces unexpected accidents such as nozzle blockage that may interfere with the manufacturing process. Therefore, the needleless electrospinning patent was invented by Jirsak in 2005 [90]. This technique loads extremely high voltages as a fiber generator and relies on the free surface of the liquid or designed bumps to excite a large number of jets simultaneously. Compared with the conventional method, the needleless electrospinning significantly improves the production rate (from 1 to 5 ml·h^−1^ [91] to 18 ml·h^−1^ [92]). Jiang et al. [93] proposed a needleless electrospinning technique that uses a stepped pyramid-shaped spinneret, with the jets generated from the edge of the steps (Fig. 6A). The spinneret system used in this technique can be divided into stationary and rotating spinnerets [94]. In needleless electrospinning technology, the spinneret system can be categorized into stationary and rotating spinnerets. When using a stationary spinneret, protrusions on the spinneret surface can be generated by applying external forces (such as magnetic fields [95], electric fields [96], or airflow [97]) to create a Taylor cone. The rotating spinneret generates multiple jets by applying a sufficiently high voltage. Various shapes like discs, cylinders, and spheres have been used in rotating spinnerets [96,98]. Upon the combination of these spinnerets, multiple jets can be generated simultaneously, which greatly improves production efficiency.

Centrifuge electrospinning is another novel electrospinning technology that uses centrifugal force to achieve massive manufacturing of electrospun fibers. The principle is analogous to the cotton-candy-making process. The whole instrument uses a transparent goalkeeper seal. During the electrospinning process, the cylinder is spun to generate centrifugal force, the polymer solution is sprayed from the nozzle, and the nozzle and collector rotate at the same speed, thus adjusting the diameter of the nanofibers by controlling the rotational speed [99]. Compared to traditional electrospinning, the nanofibers produced by this technique are more uniform and can produce finer nanofibers. Zheng and his coworkers [100] manufacture porous carbon fibers and serve as capacitors by centrifuge electrospinning (Fig. 6B). It has been well evaluated as a material for supercapacitors. This flexible wearable material provides a new possibility for the future energy storage device.

## Electrospinning for Precise Medicine

The emergence of the above-summarized advanced electrospinning techniques has realized the manufacturing of nano/microfibers with a variety of novel functionalities, promoting the development of biomedical devices for precision medicine. This section focuses on the impacts of advanced electrospinning techniques on improving the functions and performance of medical microrobots, biosensors, and OOCs.

### Electrospinning for medical microrobots

The medical microrobot, a miniaturized device with its size at the nano/micrometer scale, is capable of moving in a fluid under the activation and navigation of an external stimulus, such as physical stimulation (e.g., magnetic fields [7,101], acoustic fields [102], and light), chemical energy (e.g., bubble-induced driving force generated from the decomposition of hydrogen peroxide) [103], or a combined actuation approach [11,104–106]. The locomotion behaviors (e.g., movement direction, velocity, and precision) of the microrobots are usually controllable by adjusting the actuation parameters, which provides a miniaturized mobile platform in precision medicine applications, such as targeted drug delivery, detoxification, microsurgery, and disease diagnosis. To achieve targeted therapy and less/noninvasive microsurgery for precise medicine, the microrobots should possess specially designed structures and functions to realize complex locomotion, sensitivity, and responsiveness toward clinically applicable stimulus, and the possibility of high-throughput manufacturing for applications.

#### Microrobots with biomimetic structures

In comparison with the conventional designs, such as spherical, linear, and tubular shapes, the microrobots with bioinspired structures (e.g., flagellum of microorganism and tails of sperm) can perform complex movements in the viscose fluidic environment of human body by emulating the behaviors of their counterparts. To build the microrobots with complex architectures, photopolymerization technology [107], biological template deposition method [108], template-assisted electrochemical deposition [109], and glancing angle deposition [110,111] are the commonly applied techniques. However, photopolymerization technology requires specialized and expensive equipment (e.g., a 2-photon lithography system). The uniform coverage of magnetic materials on the surface of the template material is an urgent problem to be solved by the template method [112]. Both template-assisted electrochemical deposition and glancing angle deposition require professional technicians [113,114]. In addition, all the technologies above face high costs and the lack of high-throughput manufacturing.

**Fig. 7.**
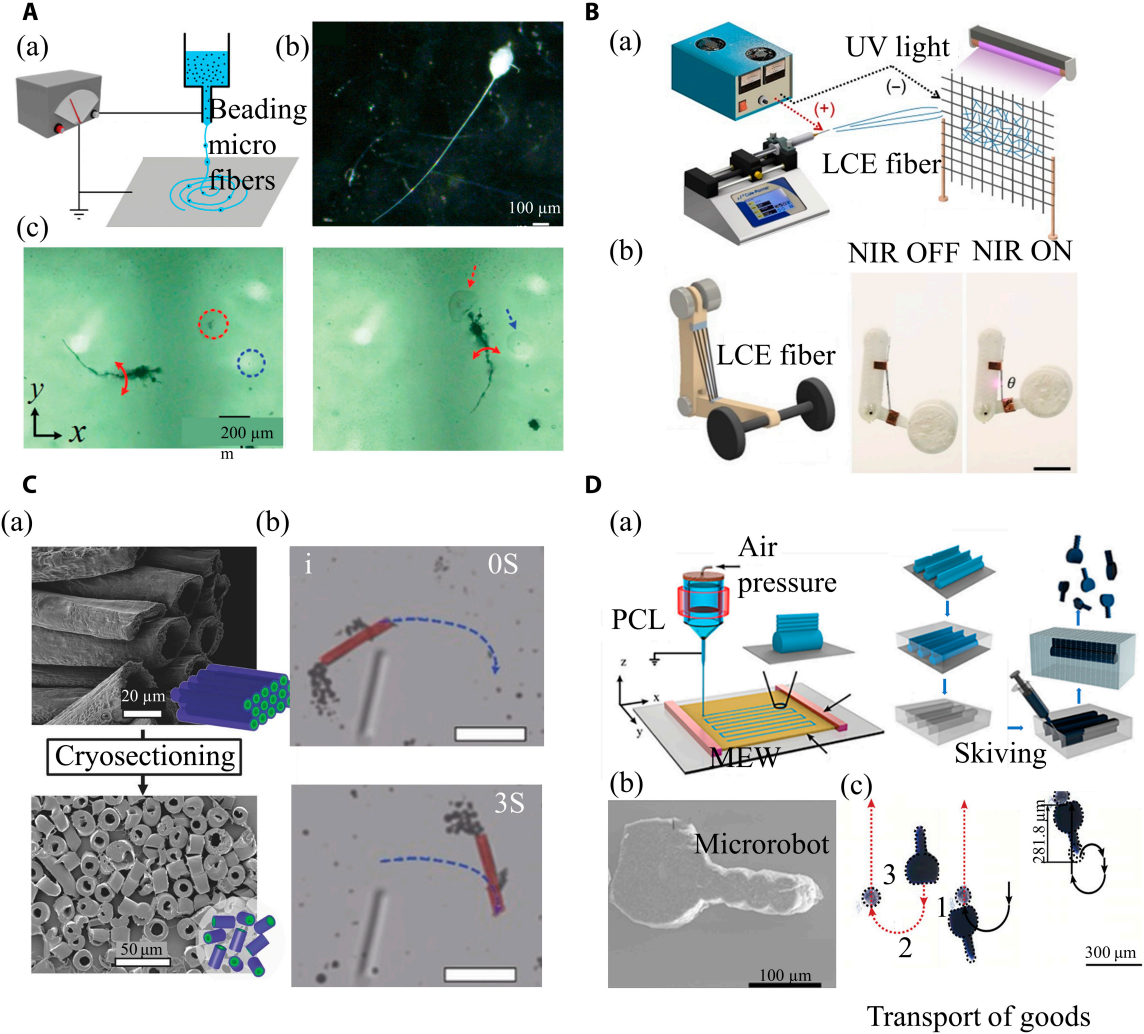
Electrospinning nanofibers in microrobots. (A) Biomimetic and magnetic microrobots. (a) Diagram of manufacturing the sperm-like microrobots. (b) Image of the sperm-like microrobot. (c) The motion of microrobot in the magnetic field (2 Hz). Reproduced with permission from [115]. Copyright Rights managed by AIP Publishing. (B) Light-driven LCE microfibers. (a) Electrospinning of LCE microfibers. (b) Schematic of lifting arm constituting LCE fibers and the principle of their light-driven deformation. Reproduced with permission from [123]. Copyright 2021 The American Association for the Advancement of Science. (C) Electrospinning for microscale rockets. (a) SEM image of PLGA/PEO hollow fiber (top) and 10-μm microtubule (bottom). (b) Movement of the microscale rockets in H_2_O_2_ concentrations (red, microrockets; dark gray, oxygen bubbles; blue, trajectories). Reproduced with permission from [133]. Copyright 2016 WILEY-VCH Verlag GmbH & Co. KGaA, Weinheim. (D) Melt electrospinning of magnetic microrobots. (a) Diagram of manufacturing the tadpole-like microrobots. (b) SEM image of tadpole-like microrobot. (c) The movement of tadpole-like microrobots carrying microballs. Reproduced with permission from [140]. Copyright 2021 The Authors. Advanced Science published by Wiley-VCH GmbH.

The electrospinning technique has demonstrated its merits in engineering biomimetic microrobots. For instance, resembling the characteristics of flagellum, the controlled whipping of electrospun nano/microfibers can be utilized to directionally propel the cargos at a desired velocity in liquids. Khalil et al. [115] utilized the beads effect during the electrospinning process, an undesired phenomenon that results in in fibers with periodic or irregularly spaced spherical shapes, to produce sperm-mimicking structures composed of a head (beads) and a tail (electrospun homogeneous fiber). The sperm-like microrobots can replicate the motion of a typical flagella swimming (200 μm·s^−1^) under the magnetic fields (2 Hz) (Fig. 7A). In comparison with the conventional microrobots with regular shapes, such a biomimetic structure of the robot can improve its mobility by emulating the motions of cells or microorganisms. For example, the flagellum movement of the sperm-like microrobots can realize the active reverse blood flow using the flagellum movement to achieve targeted destruction of the lesion site. Through the drug loading on the sperm head, targeted drug delivery (such as heparin [116] and anticancer drugs [117]) can be achieved. However, rather than generating smooth fibers, the bead effect of the electrospinning is usually irregular that is influenced by the solution property, electrospinning parameters, and ambient conditions. Therefore, defining and controlling the dimension and structure of the electrospun sperm-like microrobots remain challenging.

#### Adaptivity to diverse actuations

With the emergence and advances of medical technology and equipment (e.g., magnetic resonance system, ultrasonic device, and fiber optic system), the magnetic, acoustic, and optic fields can be precisely controlled, enabling the rapid development of physically actuated microrobots [118,119].

The use of magnetic fields in the manufacture of magnetically driven microrobots for precise medicine is highly favored because of their strong penetration ability, good controllability, and long driving distance [120]. Electrospinning technology has also seen many effective applications in the fabrication of these magnetically driven microrobots. Achieving independent movement of multiple microrobots in a magnetic field is a challenging aspect of magnetically driven microrobots. Khalil et al. [121] applied electrospinning to create 2-tailed microrobots with a tail length ratio of 1.75, which could move at speeds up to 60 μm·s^−1^ under a 2-Hz magnetic field. Utilizing varied reversal frequencies, the microrobots with different tail length ratios achieved independent movement in an identical magnetic field. This established a foundation for controlling complex motion patterns of multiple microrobots. In addition to fulfilling the controlled movement of multiple microrobots, the challenges of ultralong-range magnetic actuation and precise navigation under a magnetic field also hinder the development of magnetically driven microrobots. Wei et al. [122] fabricated dual-layer hydrogel soft microrobots comprising a prepolymer of copolymer of 4-acryloyl benzophenone and *N*-isopropylacrylamide layer and a Fe_3_O_4_/polyacrylonitrile (PAN) layer composite through electrospinning. These microrobots were capable of precise directional navigation under magnetic field control, and the material compositing achieved through electrospinning endowed the robots with high tensile strength (4.59 MPa), which might open a new avenue for constructing multifunctional robots using electrospinning.

Light is another useful propulsion resource for actuating microrobots. Light-driven microrobots need to possess photosensitive properties and mobility based on light-induced deformation or light-thermal reactions. The great adaptability of electrospinning to various materials makes the manufacturing of electrospun light-driven microrobots feasible. He et al. [123] reported the fabrication of a liquid crystal elastomer (LCE) microfiber using electrospinning technique that can be actuated by near-infrared (NIR) laser irradiation. For the small dimension (10 to 100 μm in diameter), the LCE microfibers can generate large actuation strain (approximately 60%) with a fast response speed (less than 0.2 s) and a high power density (400 W·kg^−1^) (Fig. 7B). Using this type of fiber, a light-driven microtweezer and a microrobot were fabricated, which can technically meet the operational needs of microsurgery. On the other hand, the light-driven microrobot could achieve rapid displacement based on large-range strain. Feng et al. [124] manufactured a light-driven deformable PVA/carbon micromotor using electrospinning, which exhibits rapid moving speed (39 mm·s^−1^), directionally controlled, and multimodal locomotion (vertical, horizontal, and rotatory motions). Although these studies have successfully engineered light-driven microrobots and verified their motility in vitro, the low penetration depth of lasers (a few millimeters) through human tissues remains a substantial challenge for the application of microrobots in the human body.

Ultrasounds have been widely used in the medical field due to the deep penetration and the safety of the human body. Research has already achieved targeted drug delivery using ultrasound-driven microrobots, proving the feasibility of ultrasound-driven robots in precision medicine [125,126]. Nevertheless, there has not yet been research on manufacturing ultrasound-driven microrobots using electrospinning technology. Nonetheless, there have been plenty of reports applying electrospinning technology to fabricate acoustic-sensitive nano/microfiber using smart materials such as polyvinylidene fluoride [127,128] and piezoelectric ceramics [129]. Hence, it would be possible to engineer acoustic-driven medical robots in the near future.

Besides the physical stimulations, chemical energy derived from surroundings can also be converted into mechanical energy, enabling the movement and function of designed microrobots. The elaboration on the structure and composition design of the electrospun microrobots can enable the conversion of chemical energy into driven forces by chemical reactions [130]. The most common method of chemical driving is to generate bubbles through the reaction of hydrogen peroxide. Several pioneering studies have demonstrated the feasibility of this approach for propulsion [131,132]. In a representative study, coaxial electrospinning was used to fabricate a rocket-like microrobot (mean diameter of 16.5 ± 2 μm) composed of PEO and poly(lactic-*co*-glycolic acid) (PLGA) and modified it with metal particles (e.g., platinum, nickel, MnO_2_, or silver) or catalase as catalyzers for the initiation of the disproportionation of H_2_O_2_ to water and oxygen reaction, which propels the microrobot to move (in a range from 69 to 102 μm·s^−1^) (Fig. 7C) [133]. Because of the uncontrollable chemical reaction process in vivo, chemically actuated microrobots may suffer from intractable moving speed and navigation. Meanwhile, the chemical reaction substrate or product might lead to adverse effects on the human body, and the application of chemical-driven microrobots to clinical scenarios remains challenging. Future works should focus on the design and manufacturing of microrobots with advanced microstructures with a sufficient load of catalyst to define the motion of robots, as well as seeking a mild chemical reaction to ensure human safety for the consideration of Food and Drug Administration approval.

Utilizing the activity of cells or microorganisms (e.g., contraction and chemotaxis) is another effective method to biologically actuate the microrobots. Using living cells as the engine of microrobots can address the issues of energy efficiency and poor biocompatibility associated with external driving forces and effectively avoid the harm external drivers that can cause to the human body [134]. Successful construction of 3D structures carrying cells through 4D printing has proven cell-driven approaches feasible. Microstructures incorporating cells have been fabricated and applied in the field of biomedical engineering [135–137]. With the development of CE technology, living microfilaments carrying cells can be produced. Wu et al. [138] used coaxial electrospinning to create hollow microfibers from PVA material containing pheochromocytoma 12 cells for directed neuronal cell culture. Approximately 40% of the microfibers were filled after 1 day of culture, and directed growth of neuronal cells along the microfibers was observed within a week. After 14 days, connections between neurons were successfully formed. Similarly, Dawson et al. [139] through electrospinning of poly(ethylene glycol) methacrylate precursor polymer fibers directly loaded C2C12 myoblasts into the fibers. The cell survival rate exceeded 85% over 14 days, providing a pathway for simulating multilayered tissue structures and functions, demonstrating the capacity of electrospun fine fibers for guiding cellular functions. Despite the successful electrospinning of cell-laden microfibers, relevant reports regarding the biologically actuated microrobots are still under expectation. The design of structures to fully utilize the intrinsic power of cells (e.g., isotropic cellular alignment for maximizing linear contraction) is a consideration for the manufacturing of biomicrorobots. Moreover, controlling the movement direction of simple cell-carrying microrobots is challenging. Incorporating magnetic particles into the robot is an effective driving method. Besides, the maintenance of cellular viability and functional stability should be taken into account for their viability in the human body.

#### High-throughput manufacturing of microrobots

Another merit of electrospinning is the ability of massive manufacturing for industrial production at a low cost, which holds great promise for realizing the scale-up fabrication of microrobots. Su et al. [140] realized the large-scale fabrication of tadpole-like magnetic microrobots based on the combination of MEW and microprocessing techniques (Fig. 7D). Such a design enables 2 motion modes of the microrobots, rolling (2 mm·s^−1^) and propulsion (0 to 340 μm·s^−1^). The microrobot permits to mobilization of the objective cargo. The integration of MEW and micromachining technology greatly reduces the manufacturing cost of microrobots and provides a method for the massive manufacturing of microrobots. The precise navigation of robots under magnetic fields shows great potential for the local delivery of drugs and cells for target therapy, as well as the removal of pathological substances such as blood clots.

Apart from such a strategic approach, the production of abundant fibers has been achievable using advanced techniques (e.g., needleless and centrifugal electrospinning). As the structural and compositional designs of microrobots are optimized on the basis of the pioneering efforts, it would be possible to realize the scale-up fabrication of functional microrobots with industrial scalability.

### Electrospinning for biosensors

Because of the advantages in miniaturization, wearability, and real-time monitoring, biosensors have greatly promoted the development of efficient, accurate, and timely diagnostics in precision medicine [141]. Biosensors typically consist of a bioreceptor, a transducer, and a converter. To precisely detect the physiological and pathological changes in the human body, the bioreceptor should be highly sensitive to identify minute changes in monitored signals. For wearable and implantable biosensors, on the other hand, the materials must be biocompatible and compliant with the movement modes and tissue modulus [142,143].

Advanced electrospinning technology can not only create ultrafine micro/nanofibers but also enable flexible definitions in terms of composite components and fiber arrangement. These advantages allow for the creation of metamaterials, a class of artificial materials engineered to have properties that are not found in naturally occurring materials, which exhibit unique performances in terms of electromagnetic sensitivity, mechanical properties, and biofunctionality [144]. Therefore, electrospinning has been widely used to explore a variety of advanced biosensors.

#### High-sensitive electrospun biosensors

As an essential element, the electrospun membranes have been used in a variety of biosensors, including piezoelectric, electrochemical, and optical biosensors to measure physiological parameters such as blood pressure and body temperature. The membranes made from electrospun nanofibers typically have a high specific surface area and volume ratio, which allows the immobilization of a tiny amount of biomolecular on the sensor platform. Besides, the large specific surface area of electrospun fibers facilitates the contact between sensors and target analytes, thereby enhancing the sensitivity of biosensors [145–147].

Piezoelectric sensors are frequently used to monitor heart rate, pulse, and limb movement. The principle is based on the change in resistance driven by the deformation of the sensor, thereby displaying changes in monitored parameters [147]. Electrospun aligned fibers can greatly improve the piezoelectric capabilities of piezoelectric sensors, thereby enhancing their sensitivity. Chen et al. [148] invented a versatile biostrain sensor applicable for human motion detection, temperature monitoring, and respiratory monitoring. Because of the aligned arrangement of its electrospun ionic liquid/thermoplastic PU (TPU) nanofibers, enhanced ionic conduction is achieved, resulting in high sensitivity (2.75%·°C^−1^), a wide strain range (0% to 200%), and a low detection limit (0.1%). Similarly, Li et al. [149] utilized the ordered structure of electrospun conductive composite fibers to create highly sensitive flexible strain sensors. The tensile sensitivity perpendicular and parallel to the fiber alignment reached 0.73 and 0.01, respectively. Besides changing fiber alignment, utilizing composite materials to enhance piezoelectric performance is also a method to increase the sensitivity of piezoelectric sensors. Liu et al. [150] achieved the fabrication of a highly sensitive pressure sensor (7.7 Pa^−1^) through electrospinning fibroin proteins/graphene nanofiber membranes (NMs), which is realized by the good electrical conductivity of graphene materials and the 3D structure of conjugated electrospinning. Liu et al. [151] successfully improved the dispersion of BaTiO_3_ in the matrix, effectively enhancing its piezoelectric performance (>200%) by incorporating MXene into a BaTiO_3_ and poly(vinylidene fluoride-trifluoroethylene) matrix and subsequently electrospinning the mixture.

Electrochemical biosensors are mainly composed of a biomolecular recognition element and an electrochemical transducer. Enzyme-based biosensors are a common type among electrochemical biosensors, which achieve high sensitivity and specific recognition through the specific sensitive recognition of the target product by the enzyme, a common means to detect certain diseases (such as diabetes and cancer). The core–shell fibers are capable of loading biomolecules such as enzymes within the membrane, which avoid structural damage while enabling the specific immune identification of biological substances in the human body [152]. Ji et al. [153] used coaxial electrospinning to fabricate a glucose biosensor with a PU hollow NM in which 2 types of enzymes, glucose oxidase and horseradish peroxidase, were fixed. The glucose biosensor showed a rapid linear response in the range of glucose concentration from 0.01 to 20 mM, covering the scope of normal glucose concentration in human serum (3.9 to 6.4 mM) [154]. Kim and Kim [155] developed a hydrogel patch capable of detecting glucose concentration. By incorporating gold nanoparticles to modify glucose oxidase, they effectively enhanced the sensor’s sensitivity to 47.2 μA·mM^−1^. Since the enzymes are intrinsically vulnerable and can be easily denatured or inactivated by alterations in pH and temperature, the stability of enzyme biosensors is poor. Therefore, the creation of enzyme-free biosensors is necessary to circumvent this issue. Xu et al. [156] utilizing electrospinning technology fabricated CuO–NiO composite fibers and used these fibers to modify glassy carbon electrodes, thereby creating a nonenzymatic glucose sensor. Through the modification of the electrode with composite fibers, the sensor achieved a high sensitivity of 4022 μA·mM^−1^·cm^−2^ and maintained long-term stability.

**Fig. 8.**
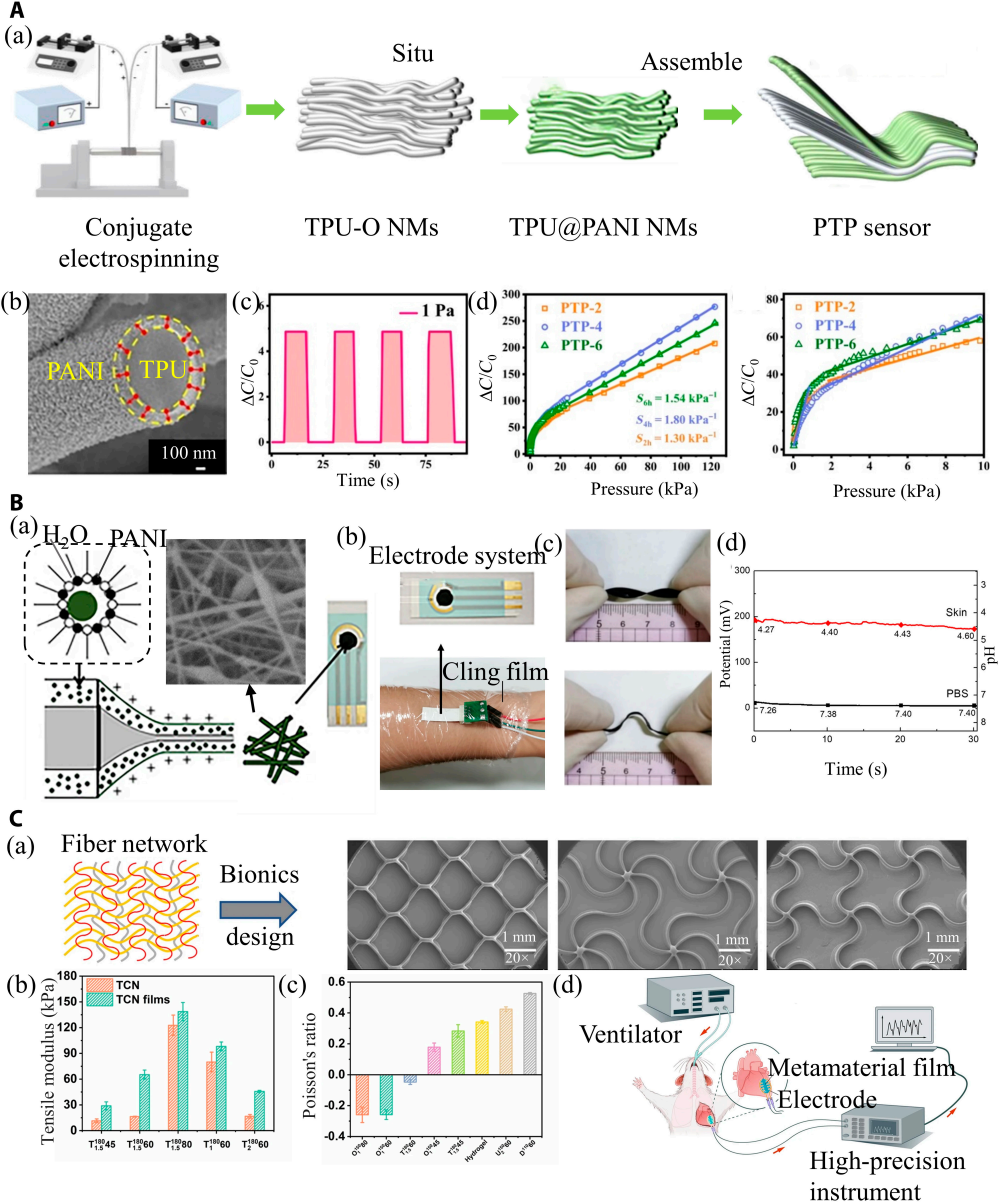
Electrospinning nanofibers in biosensors. (A) Conjugated electrospinning pressure biosensor. (a) The manufacturing process of sensors. PTP sensor: top: TPU@PANI NMs; middle: TPU-O NMs; bottom: TPU@PANI NMs. (b) Cross-section of the TPU@PANI nanofibers. (c) The minimum detection limit of the sensor. (d) The sensitivity test of the biosensor. Reproduced with permission from [157]. Copyright 2023 The Authors. Published by Elsevier B.V. (B) Coaxial electrospun flexible PANI//PU nanofibers as pH wearable biosensor. (a) Preparation of electrospun fiber membrane for pH wearable sensor. (b) The image of wearable biosensors for skin pH detection. (c) Photographs of deformation (top: twist; bottom: bend). (d) Sensors detect the sweat pH on the surface of the skin. Reproduced with permission from [158]. Copyright 2020 Springer Science Business Media LLC, part of Springer Nature. (C) Biosensor for implantable cardiac monitoring. (a) Biomimetic design of microfibers (unidirectional, triangular, and orthogonal waves). (b) The elastic modulus of the triangular corrugated networks (TCN) and TCN films. (c) Poisson's ratio of GCF at 15% strain. (d) Schematic of the metamaterial film to monitor cardiac deformation. Reproduced with permission from [164]. Copyright 2023, The Author(s).

Aside from enhancing sensor performance through material modification, directly fabricating fibers with ideal properties presents another avenue for sensor development. For instance, designed microstructures can endow the metamaterials with improved sensitivity toward specific stimuli. Jin et al. [157] prepared the orientated TPU elastomer (TPU-O) rubber NMs by the conjugated electrospinning for producing a pressure biosensor. By assembling the PANI NMs above and below the TPU-O NMs, as illustrated in Fig. 8A, a sensor called “PTP sensor” is formed. Such a PTP sensor improves the sensitivity (31.73 kPa^−1^) due to the form of a bamboo-raft-like microstructure between the aligned fibers under pressure. Meanwhile, TPU-O NMs ensure the flexibility and excellent tensile properties of the biosensor, which exhibits a minimum detection limit of 1 Pa and a good linearity in a broad working age (1 Pa to 122.5 kPa). Upon coating with PANI nanoparticles, the conductivity of the membrane was reinforced whereby permitted the biosensor to be used for the detection of human movement (Fig. 8A).

#### Wearable electrospun biosensors

To achieve real-time monitoring for precise medicine, wearable and implantable biosensors have been placed under the spotlight. Comfort, flexibility, and biocompatibility are primary requirements for wearable devices, which necessitates that the materials used in biosensors must possess excellent mechanical properties and biocompatibility.

The simplest method to enhance mechanical properties is through material compositing. Moreover, core–shell fibers produced by coaxial or emulsion electrospinning can be applied to manufacture the composite membrane for improving the mechanical properties and electrical conductivity of biosensors. Hou et al. [158] used coaxial electrospinning to fabricate a composite acid–base sensor made of PU and PANI (in the pH range of 2 to 7) (Fig. 8B). The involvement of PU improves the mechanical properties of the sensor which can resist twist, bending, and stretching deformation to 250% in the dry state. It is expected that the electrospinning of PU/PANI composite fibers may pave the way to develop wearable biosensors due to the directed structural combination of conductive and flexible material.

Upon the electrospun flexible structures, additional conductive components such as metallic and carbon materials can be further involved to construct the biosensors with excellent flexibility and high sensitivity. As a representative flexible material, TPU is widely applied for manufacturing wearable devices. Wang et al. [159] created a motion sensor capable of 200% deformation by coating TPU fiber membranes with liquid metal. The excellent flowability, low viscosity, and low toxicity of liquid metal allowed for the homogeneous coverage of the TPU membrane, which significantly enhanced both the stretchability and sensitivity of the sensor. Zhao et al. [160] immersed electrospun TPU membranes in Ti_3_C_2_ to fabricate sensors for human motion monitoring, extending the strain range to 300%. Besides TPU, polydimethylsiloxane (PDMS) is another commonly used flexible material, and studies have shown its feasibility in flexible, wearable devices [161,162]. Therefore, relying on the material adaptability of electrospinning technique, it would be possible to engineer a variety of wearable biosensors by altering the material selection according to the specific application scenario.

#### Implantable electrospun biosensors

Despite the wearable device can monitor physiological data in a noninvasive manner, its accuracy significantly varies depending on the device’s quality and the sensor’s placement. In comparison, the implantable biosensors that are surgically placed in affinity of targeted tissue/organ can provide continuous, real-time monitoring of health parameters with high precision. Conversely, because of the direct contact with tissue/organ, the implantable biosensors should be mechanically conformality to avoid adverse effects (e.g., inflammation and hyperplasia) caused by the mismatched motion and modulus with that of tissue [163]. Besides, similar to the requirement of tissue engineering products, the sensor should be biocompatible and/or biodegradable to prevent body rejection and facilitate the removal after completing its mission.

Facing these considerations, electrospinning of biometamaterials has been designed for the implantable sensors. For instance, gelatin, one of the most commonly used biomaterials with excellent biocompatibility, is an ideal choice for implantable sensors. However, the low toughness and high elasticity (>1 MPa) of gelatin are contrary to the needs of human soft tissues (such as cardiac; the elastic modulus of the heart is 5 to 50 kPa), limiting its applications in engineering implantable sensors. To solve this problem, Chen et al. [164] followed the design concept of metamaterials and applied MEW to develop a unique gelatin-based conductive film (GCF) by embedding ultrafine fiber networks into a hydrogel with an ultralow elastic modulus. This innovative GCF is characterized by its mechanical programmability, enabling it to mimic the mechanics of soft tissues with an elastic modulus ranging from 20 to 420 kPa and a Poisson’s ratio extending from −0.25 to 0.52. The negative Poisson’s ratio is a critical feature, enhancing the film’s compatibility and conformity with soft tissues, thereby improving the efficiency of biological interfaces. The GCF’s high conformability makes it particularly adept at monitoring physiological signals such as heartbeat and respiratory rate, achieved by tracking cardiac deformations (Fig. 8C) [164].

A significant advantage of this gelatin-based film is its biodegradable nature, allowing not only for monitoring but also for supporting tissue restoration. This dual functionality underscores the potential of the GCF in bioelectronic applications, particularly in developing implantable sensors that synergize monitoring with tissue repair. Furthermore, the metamaterial design of the GCF opens new avenues in the field of bioelectronics, especially for creating implantable sensors. These sensors are tailored for high conformity with soft tissues, providing a customized approach to enhancing the functionality and efficiency of implanted bioelectronic devices. This innovation based on the integration of electrospinning technique and biometamaterials design may bring the advent of advanced and effective implantable sensors for biomedical applications.

Collectively, the advanced electrospinning techniques have demonstrated their ability to promote the selectivity, sensitivity, flexibility, and viability of biosensors. Even so, because of the complexity of applied advanced electrospinning techniques, the scale-up manufacturing of relevant product remains challenging. To this end, the integration of high-throughput manufacturing approaches (e.g., centrifuge and needleless electrospinning) may offer a solution to translate the technique from laboratory to industry [165]. For instance, Jiang and Qin [166] developed a novel coaxial electrospinning process modified upon the free surface electrospinning technique that can drastically increase production efficiency by more than 100 times compared to conventional coaxial electrostatic spinning.

### Electrospinning for OOCs

OOCs are capable of offering a 3D microenvironment that resembles the physiological conditions for the in vitro culture of human cells, which thereby can reflect the fundamental function and responses of human tissues or organs, as well as the correlations between different tissues/organs [167–170]. Because of this superiority, the OOCs are considered one of the most innovative biomedical devices that can replace or correct animal models and planar cell models to undertake preclinical studies for the discovery of new drugs [171].

 OOCs typically consist of multiple houses partitioned by permeable membranes and microfluidic channels for the purpose of accommodating different types of cells/tissues to emulate the tissue interfaces and supplying metabolic needs and physiological signals (e.g., flow, pressure, and gradients) to the coculture system, respectively. Since acquiring functional tissues is a fundamental requirement of OOCs, each element should be carefully designed and fabricated to promote cellular activities, cell–cell interactions, and tissue morphogenesis.

#### Weaving functional membranes for OOCs

As an essential component of OOCs devices, the membrane not only serves to separate the microfluidic channels for the mimicry of tissue/tissue interface but also provides a biological substrate for supporting cell activities. Therefore, an ideal membrane should possess appropriate mechanical strength to resist the applied dynamic flows and pressures, ECM-mimicking compositions (e.g., laminin and type IV collagen in basement membrane) that promotes cellular functions and tissue morphogenesis, and sufficient porosity that confines cell transmigration but permits cellular cross-talk. To this end, electrospinning exhibited superiority to other approaches due to the ability to produce the membranes with predesigned properties upon the braiding of bioactive, orchestrated, or heterogeneous nano/microfibers.

Porous PDMS sheets are the most commonly used membranes for OOCs devices due to the high transparency for real-time monitoring, acceptable air permeability for cell culture, and good elasticity to endure the deformation caused by the given dynamic physiological signals. Man et al. [172] electrospun PDMS fibrous membranes for coculture of epithelial cells with fibroblasts embedded in 3D collagenous gels. The robust mechanical strength of PDMS membrane allowed for the imposition of interstitial fluid flow and 3D breathing-like mechanical stretch to the OOCs system, replicating the the key anatomical and physiological characteristics of human alveoli. Compared with the transwell model, OOCs significantly improve the function of the epithelial barrier. The permeability of dextrans through the cells grown in OOCs is about 3 to 4 times lower than that in the transwell model. Despite the important merits of PDMS, the lack of sufficient bioactivity limits the adhesion and growth of cells.

**Fig. 9.**
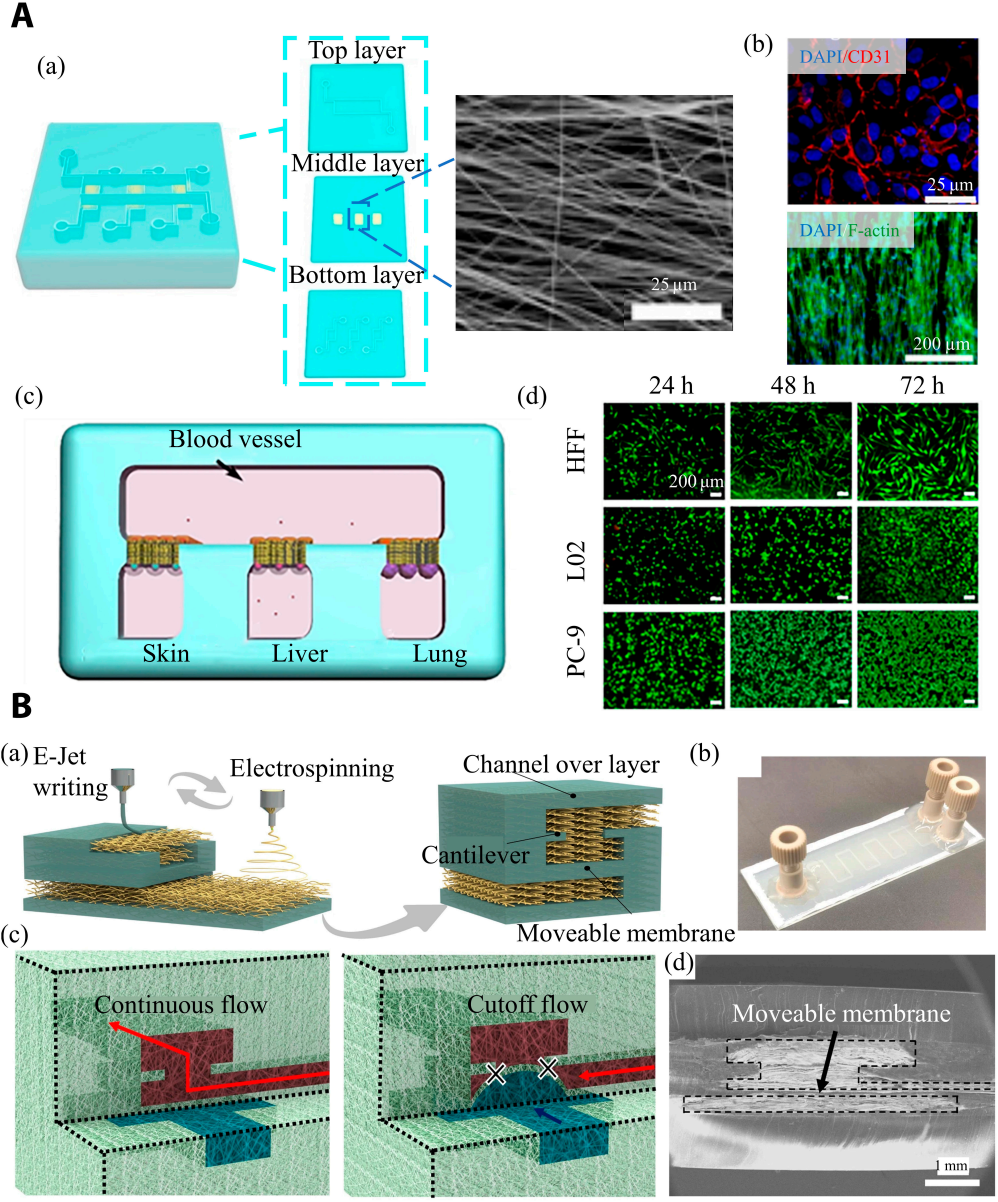
Electrospinning nanofibers in OOCs. (A) Multicompartment OOCs. (a) The structure of the OOCs and images of OOCs electrospinning NMs. (b) Top: CD31 expression of HUVECs; bottom: fibroblast orientation. (c) Construction of jaundice model in vitro with different OOCs (vessel/skin/liver/lung). (d) Cell culture of human foreskin fibroblast (HFF), hepatocytes (L02) and lung cells (PC-9). Reproduced with permission from [174]. Copyright 2021 Donghua University, Shanghai, China. (B) Electrospinning and electrohydrodynamic jet (E-jet) writing manufacture 3D microfluidic chip. (a) Schematic diagram of microchannels manufacturing. (b) Images of the microfluidic chip with single-layered (263 μm in width and 56 μm in height). (c) Schematic diagram of microfluidic pressure valve. (d) Cross-section image of the valve. Reproduced with permission from [186]. Copyright 2022 The Author(s).

To improve the biological function of the membranes for OOCs, material modification is one adaptable choice. For instance, PCL owns strong mechanical properties and good biocompatibility, but the hydrophobicity confined its cellular affinity. On the other hand, abundant active groups presenting in collagen facilitate cell adhesion and proliferation. The incorporation of natural polymers into synthetic materials has been widely used to balance the mechanical and biological properties of electrospun products. Kanabekova et al. [173] developed PCL–collagen fibrous membranes for lung OOCs. Compared with pure PCL membranes, the Young’s modulus increased by 15 times, and cells within the chip exhibited nearly equivalent biological activity to that in ex vivo membrane cultures. Through computational fluid dynamics, the optimal conditions for the use of electrospun fibrous membranes were determined (range of physiologically relevant shear stress is 0 to 0.03 dyn·cm^−2^), which have a positive effect on promoting cell growth and functional expression on OOCs. Lei et al. [174] electrospun poly(l-lactic acid) (PLLA)/collagen I composite nanofibers to weave membranes for the construction of jaundice disease models by creating skin, vessel, liver, and lung OOCs. The PLLA/collagen I NM endows tissue–tissue interfaces with excellent semipermeability, appropriate mechanical support, cell orientation induction, as well as good cell adhesion and proliferation capabilities. The fluid velocity and shear stress on both sides of the membrane are basically consistent with that of natural blood vessels. This indicates that the collagen and PLLA blended membrane can combine the advantages of biocompatibility and mechanical properties, pointing out a practical and feasible manufacturing method for constructing OOCs membranes (Fig. 9A) [174]. The functional membrane showed unique features, including excellent biocompatibility, strong cellular affinity, controllable orientation, tunable thickness, and porosity, which endow the tissue–tissue interface with excellent semipermeability, appropriate mechanical support, inducible cell orientation, good cell adhesion, and proliferation.

Besides the hybridization of multiple types of materials, heterogenous fibers (bioactive materials/synthetic polymers) and orchestrated fibers produced by advanced electrospinning techniques may achieve an identical objective by encouraging cellular functions and guiding cellular arrangement/migration, respectively. Despite the few reported studies, the structures composed of core–shell or Janus fibers, as well as aligned fibers, have been utilized as biofunctional scaffolds for tissue regeneration and repairs (e.g., blood vessel grafts [175,176], skin grafts [177,178], and cardiac patch [179,180]), demonstrating their potential to be advanced membranes in OOCs devices.

Besides the bioactivity, the design of membrane’s microstructures also plays an important role in engineering desired functional OOCs, including the porosity and pore size. Sufficient porosity allows for paracrine signalings and penetration of metabolic products. On the other hand, while the large pores (dozens or hundreds of micrometers) facilitate the infiltration and growth of seeded cells, the tiny pores (micro/nanometers) serve to prevent the migration of cells into undesired regions [181]. Therefore, the porous membrane with varied pore sizes is necessary for OOCs. Tuerlings et al. [182] established a human osteochondral unit on-chips based on a structurally composite PCL membrane that is composed of 2 distinct layers. A microfiber layer, with an average thickness of 190.1 ± 30.58 μm, fiber diameters around 8.60 ± 0.97 μm, and pores averaging 25.51 ± 12.37 μm, was used as a scaffold for loading primary osteogenic cells. Upon it, a nanofiber layer characterized by finer fiber diameters of 0.74 ± 0.55 μm and smaller pores of 2.14 ± 1.14 μm functions to prevent these cells from migrating to the compartment housing human primary articular chondrocytes. After culturing for 3 weeks, bone- and cartilage-like tissue was generated on the platform that enabled the creation of the osteoarthritis disease model. To recapitulate the physiological features and interactions between multiple tissues/organs using the OOCs, both functional tissue and tissue–tissue interfaces should be recreated. The concept of using the electrospun membrane with varied microstructures provides an optional way to construct the OOCs while accommodating multiple types of tissues.

In addition, it is also worth noting that the electrospinning technique not only contributes to producing functional membranes but also opens an avenue for manufacturing the OOCs. In particular, the membrane can be easily integrated into the processes for the construction of OOCs devices due to its advantages of simple and low-cost features. Johanna et al. [183] directly deposited electrospun membranes with different compositions onto the microfluidic channels, creating a versatile fabrication method for the construction of OOCs platforms that can be tailored to possess tissue-specific microenvironments. Such direct membrane process and integrating method also help to improve the quality of OOCs. In a recent study, Rhyou et al. [184] introduced a facile fabrication process of an electrospun NM-integrated PDMS microfluidic chip. Their results indicated that the uncured PDMS adhesive layer in the chip maintains the smooth surface after electrospinning and allows the rapid and leakage-free bonding of the chip using plasma treatment. Therefore, fully utilizing the flexibility of electrospinning techniques may accelerate the advent of multiple OOCs or human-on-chips.

#### Creating microfluidic channels for OOCs

The OOCs are basically microfluidic devices. Diverse approaches have been utilized to create microchannels, including lithography, precise etching, micromolding, and 3D bioprinting techniques. Nevertheless, these technologies are limited either in complex procedures and high costs or in the construction of complicated structures with biomaterials that intimate the complexity of natural counterparts (e.g., vascular networks). For instance, the lithography and precise etching technique usually rely on sophisticated systems and a series of steps to build the microchannels embedded in PDMS, plastics, and glasses that are difficult to reflect the mechanical features and biological functions of ECM in the human body. On the other hand, molding and 3D printing methods are capable of creating microchannels in biocompatible hydrogels but are limited to engineering channels with intricate architectures or ultrafine dimensions.

The fibers with tunable dimensions and aligned patterns can be readily fabricated using advanced electrospinning techniques, such as near-field and melt electrospinning. The precisely orchestrated fibers with hierarchical sizes (from tens of micrometers to nanometers) can play a role as templates of microfluidic channels, which provides a simple but versatile manner to construct the OOCs devices. Zeng et al. [185] created PDMS microchannels based on MEW. They manufactured a PCL mold using MEW and then cast PDMS over it. After removing the PCL, PDMS microchannels were obtained, with diameters ranging from 3.8 to 24.8 μm and heights from 35.3 to 225.4 μm. Subsequently, they constructed T-shaped and cross-shaped microfluidic devices, validating the feasibility of using PDMS for microfluidic channels. Apart from using electrospun molds to create microchannels, the direct construction of microchannels can also be achieved through the alternate arrangement of electrospun membranes and fibers. Qiu et al. [186] successfully fabricated 3D microfluidic structures by alternating electrospinning and electrohydrodynamic jet writing, which avoided the collapse of the 3D structure and the residual of sacrificial materials (Fig. 9B). In addition, this device can function as a valve through pressure control. On this basis, it can facilitate specific culturing in different regions of the OOCs, contributing to the construction of OOCs systems. This valve mechanism allows for precise control of fluid flow and the creation of distinct microenvironments within the chip, mimicking the complexity of human organs and tissues. Such a feature is essential for replicating physiological conditions and studying organ interactions, drug responses, and disease mechanisms in a controlled setting. Nie et al. [187] combined 3D printing and near-field electrospinning techniques to create a vascular system chip that contains both large blood vessels and capillaries. On the basis this device, the vascular-tumor coculture model is engineered as a potential preclinical tool for screening the safety and therapeutic effects of cancer drugs. A main challenge in the field of tissue engineering is the construction of a hierarchical vascular system in vitro to supply nutrients and oxygens to bulk artificial tissues and organs. Therefore, the achievements realized by advanced electrospinning techniques both enable the production of complex OOCs devices and facilitate the development of regenerative medicine.

The engineered microchannels are generally designed to transport nutrients and oxygens to cells. During the cultivation process, the density of formed tissue will elevate as a function of continuous cell growth and assembly, which limits the penetration of supplies, resulting high risk of tissue necrosis [188]. As the thickness of tissue increases (>200 μm), the level of hypoxia induced by the absence of microvasculatures inevitably deteriorates. Therefore, to promote viability and stability of OOCs, it is also indispensable to create microchannels within the engineered tissues [189]. It has been demonstrated that the topological cues contribute to promoting the vasculogenesis and the formation of neovasculature. Therefore, the nano/microfibers produced by electrospinning technique have been used to facilitate the generation of microvessels. Wanjare et al. [190] utilized electrospinning to create scaffolds of randomly oriented and parallel-aligned microfibers made of PCL, upon which they seeded induced cardiomyocytes. The scaffolds with parallel-aligned fibers significantly promoted the organization of microvessels along the direction of the fibers, with the proportion of oriented vessels ranging from 36% to 56%. In contrast, scaffolds with randomly oriented fibers exhibited superior microvessel formation within 4 weeks. Vascularization plays an important role not only in the construction of OOC systems but also in the development of artificial organs ex vivo. This aids in understanding diseases in different tissues, developing more effective drug delivery systems, and contributing to research in wound healing.

## Conclusion and Future Perspectives

Electrospun fibers are widely used in medical fields because of their high specific surface area and excellent performance in simulating extracellular matrices. With the development of advanced electrospinning technology, progress has been made in enhancing material diversity, optimizing material properties, and commercialization. Therefore, advanced electrospinning technology provides a practical means for the development of precision medicine, while promoting the development of the field.

Although electrospinning techniques have been utilized to engineer advanced biomedical devices, future research should focus on further exploring its potential to extend and improve its applications in precise medicine. For instance, to enable different motion modes, various shapes have been designed for medical microrobots, such as spiral, tubular, spherical, cellular-mimicking, etc. Despite this, only sperm- and rocket-like structures have been realized using advanced electrospinning techniques in limited pioneering works. Nonetheless, in combination with other fabrication technologies (e.g., 3D printing [191,192] and electrospray), it might be highly possible to expand the ability of electrospinning to build other types of microrobots, including the helix and bead, because even more complicated structures (e.g., stents and drug-laden spheres) have been previously fabricated.

Other than biomedical devices, there are a lot of development prospects in terms of tissue engineering and drug delivery that have been comprehensively reviewed elsewhere. The emergence of advanced electrospinning is expected to continuously bring in innovative approaches for the progress of regenerative medicine and modern pharmaceutics. In this review, the excellent achievements of directly engineering cell-laden biofibers and complex constructs were summarized. Although tissue engineers have tried to seed cells onto the electrospun scaffolds, the limited depth of cell penetration (<100 μm) confined the building of homogenous living constructs, which is possible to be overcome when involving cells within the electrospun fibers. Besides, in comparison with 3D bioprinted filaments (larger than 200 μm), the thin electrospun biofibers (approximately 10 μm) are conducive to guiding cell alignment and directional migration and thus can facilitate the construction of functional tissues/organs with anisotropic features (e.g., muscle and neuron).

The fabrication of ultrafine, ordered, and complex structures is the pursuit of nanoscience. Electrospinning technology offers significant advantages in achieving ultrafine fibers, constructing ordered arrays of nanofibers, and manufacturing special structures such as core–shell, Janus, and their combinations. Multichamber structures allow for the encapsulation and controlled release of multiple substances within electrospun fibers, which can greatly advance drug delivery technology [193,194]. Bead-on-string hybrids enable the incorporation of nanoparticles, microparticles, or other functional materials into electrospun fibers, providing opportunities for tailoring the performance and functionality of the fibers [60,195]. These achievements of electrospinning further promote the applications of nanoscience and nanotechnology in precise medicine.

Despite the remarkable merits, the drawbacks of electrospinning are obvious, such as relatively unstable product consistency and multiple control factors. To stabilize the electrospinning process, plenty of parameters should be systematically considered, including materials properties, solvent formulation, voltage level, nozzle gauge, materials flow rate, collection manner and distance, ambiance moisture, temperature, etc. Slight changes in each factor might lead to variations in fiber status. In addition, the use of chemical solvents not only suffers from the clinical safety examination but also inevitably limits the selection of vulnerable cargos that are beneficial for healthcare, such as proteins, chemical/thermal sensitive compounds, and cytokines, drastically hindering their applications in biomedical fields. Therefore, collaborations between materialists, chemists, biologists, engineers, and doctors are necessary to broaden its practice in precise medicine.

## Data Availability

Data of this paper are available by emailing lijinhua@bit.edu.cn.
